# Stochastic particle unbinding modulates growth dynamics and size of transcription factor condensates in living cells

**DOI:** 10.1073/pnas.2200667119

**Published:** 2022-07-26

**Authors:** Gorka Muñoz-Gil, Catalina Romero-Aristizabal, Nicolas Mateos, Felix Campelo, Lara I. de Llobet Cucalon, Miguel Beato, Maciej Lewenstein, Maria F. Garcia-Parajo, Juan A. Torreno-Pina

**Affiliations:** ^a^CFO-Institut de Ciencies Fotoniques, The Barcelona Institute of Science and Technology, Barcelona 08860, Spain;; ^b^Institute for Theoretical Physics, University of Innsbruck, Technikerstr. 21a, A-6020 Innsbruck, Austria;; ^c^Centre for Genomic Regulation (CRG), The Barcelona Institute of Science and Technology, Dr. Aiguader 88, Barcelona 08003, Spain;; ^d^Universitat Pompeu Fabra (UPF), Barcelona 08002, Spain;; ^e^ICREA-Catalan Institute for Research and Advanced Studies, Pg. Lluis Companys 23, Barcelona 08010, Spain

**Keywords:** liquid–liquid phase separation, transcription factor, Brownian motion coalescence, biomolecular condensates, single particle tracking

## Abstract

Liquid–liquid phase separation (LLPS) is emerging as a key physical principle for biological organization inside living cells, forming condensates that play important regulatory roles. Inside living nuclei, transcription factor (TF) condensates regulate transcriptional initiation and amplify the transcriptional output of expressed genes. However, the biophysical parameters controlling TF condensation are still poorly understood. Here we applied a battery of single-molecule imaging, theory, and simulations to investigate the physical properties of TF condensates of the progesterone receptor (PR) in living cells. Analysis of individual PR trajectories at different ligand concentrations showed marked signatures of a ligand-tunable LLPS process. Using a machine learning architecture, we found that receptor diffusion within condensates follows fractional Brownian motion resulting from viscoelastic interactions with chromatin. Interestingly, condensate growth dynamics at shorter times is dominated by Brownian motion coalescence (BMC), followed by a growth plateau at longer timescales that result in nanoscale condensate sizes. To rationalize these observations, we extended on the BMC model by including the stochastic unbinding of particles within condensates. Our model reproduced the BMC behavior together with finite condensate sizes at the steady state, fully recapitulating our experimental data. Overall, our results are consistent with condensate growth dynamics being regulated by the escaping probability of PR molecules from condensates. The interplay between condensation assembly and molecular escaping maintains an optimum physical condensate size. Such phenomena must have implications for the biophysical regulation of other nuclear condensates and could also operate in multiple biological scenarios.

## Significance

Living cells organize internal compartments by forming molecular condensates that operate as versatile biochemical “hubs.” Their occurrence is particularly relevant in the nucleus where they regulate, amongst others, gene transcription. However, the biophysics of transcription factor (TF) condensation remains highly unexplored. Through single-molecule experiments in living cells, theory, and simulations, we assessed the diffusion, growth dynamics, and sizes of TF condensates of the nuclear progesterone receptor (PR). Interestingly, PR condensates obey classical growth dynamics at shorter times but deviate at longer times, reaching finite sizes at steady-state. We demonstrate that condensate growth dynamics and nanoscale-size arrested growth is regulated by molecular escaping from condensates, providing an exquisite control of condensate size in nonequilibrium systems such as living cells.

Activities performed by living cells are generally achieved through the compartmentalization of their multiple components in space and time. Although traditionally cell compartments have been thought to be surrounded by membranes, recent evidence indicate that cells also organize membrane-less internal compartments through the physical process of liquid–liquid phase separation (LLPS) (1–4). LLPS creates transient chemically distinct compartments, also called biomolecular condensates, which may operate as versatile biochemical “hubs” inside the cell (1, 5). Phase separation is particularly relevant in the cell nucleus, where the condensation of numerous proteins on chromatin has been shown to regulate gene transcription and chromatin architecture at multiple temporal and spatial scales (6–8). Transcription factor (TF) condensates are proposed to regulate transcriptional initiation and amplify the transcriptional output of expressed genes (5, 7, 9–11). Yet despite its prevalence and biological significance, a quantitative determination and understanding of the biophysical parameters controlling TF condensation in the nucleus of living cells is largely missing. Moreover, several recent reports have challenged the notion that LLPS may indeed be responsible for the apparent condensate-like behavior of nuclear proteins, including theoretical models that could distinguish the nature of nuclear condensation based on single molecule–based physical observables (12–14). Hence, a deeper understanding on the origin of such events is needed.

Nuclear receptors are a family of TFs that have been widely studied as master regulators of gene transcription and genome topology in response to an external stimulus: a steroid hormone (15–17). Structurally, these TFs contain two intrinsically disordered regions that favor phase separation, the N-terminal domain and the hinge, as well as two highly structured regions: the DNA-binding domain and the ligand-binding domain (18). Ligand stimulation of several members of this family has been shown to trigger LLPS, forming nuclear condensates with different transcriptional roles (19–21). Since ligand addition allows accurate control of the onset for nucleation and condensate coarsening, nuclear receptors represent an ideal system to study inducible phase separation and to follow their temporal evolution in well-controlled and tunable experimental settings.

From the theoretical side, phase separation is usually associated with the heterogeneous mixing of two components, either by spinodal decomposition (22) or nucleation (23). In general, entropy-based models, such as the Flory-Huggins model (24, 25), have been commonly used to understand phase-separated systems in biological scenarios (26). Moreover, in recent years, several studies have addressed the temporal evolution of condensate nucleation and growth within the full complexity of living cells. For instance, it has been shown that biocondensate nucleation and coarsening can be described by different physical mechanisms such as diffusion-limited growth, Ostwald ripening, or Brownian motion coalescence (BMC) (27). The common physical property underlying these mechanisms is a dynamic power–law scaling behavior of the mean droplet sizes (27), with a final steady-state that results in a single condensate containing all phase-separated molecules. However, consistent deviations from these LLPS growing mechanisms have been also reported and attributed to the occurrence of active nonequilibrium processes within living cells, such as RNA transcription (27) or the presence of obstacles such as chromatin (28). Hence, models that can predict and/or adapt classical phase-separation properties to the living-cell context are still under development.

Here we investigate the physical properties of LLPS in transcriptional condensates of the nuclear progesterone receptor (PR) in living cells using an extensive combination of single-molecule approaches, theory, and simulations. Analysis of single PR trajectories showed a hormone-dependent bimodal distribution on the diffusion of the receptor associated with particles diffusing within and outside condensates. Using a deep-learning method, we found that diffusion within condensates is best described by means of fractional Brownian motion (29), whereas outside condensates, diffusion is anomalous and heterogeneous. High-density single-molecule localization maps as a function of time further revealed a BMC-like growth process at shorter times but one that markedly deviated at longer timescales, reaching a growth plateau on the condensate sizes at the nanoscale. To quantitatively understand our observations, we developed an extension of the BMC model by including the stochastic unbinding of particles within condensates. Our model can not only reproduce the usual BMC behavior, but notably, it also reaches a steady state with finite condensate sizes. As a whole, our single-molecule experimental data and theoretical model is consistent with droplet growth dynamics being regulated by the escaping probability of TF molecules from condensates.

## Single-Particle Tracking of Nuclear PR in Living Cells in Response to a Tunable Stimulus

**Fig. 1.**
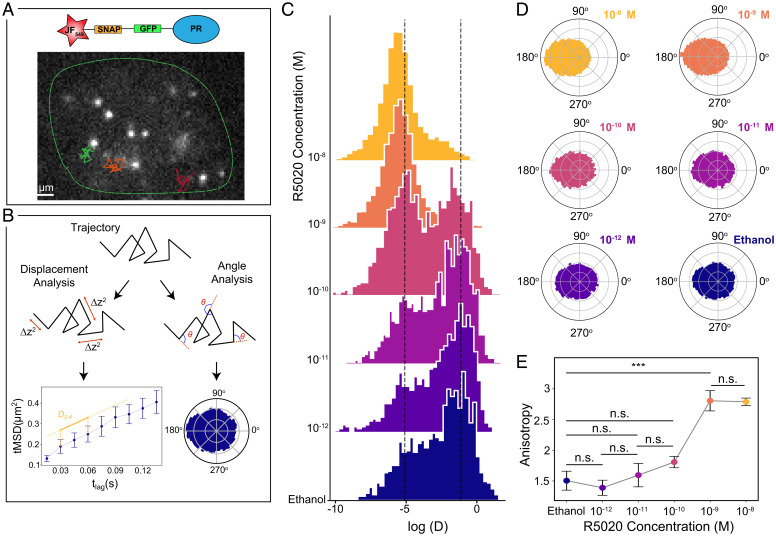
Lateral diffusion of individual PR molecules in the nucleus of living cells. (*A*) Representative frame of a SPT video. Individual PR molecules (bright spots) were visualized in the nucleus (green outline) of MCF7 breast cancer cells, under a highly inclined illumination at a 15 ms frame rate. Diffraction-limited single-molecule localizations were tracked in successive frames to generate individual trajectories (superimposed color lines). (*B*) Schematic representation of the trajectory analysis. For each trajectory, we extracted the displacement between frames to generate individual tMSD plots as a function of the time lag and extracted the diffusion coefficients (*D*_2 − 4_) for each trajectory (*Left, Bottom*) (Error bars, SEM). In addition, we calculated the angles between successive steps to create polar histograms (*Right, Bottom*). (*C*) Distribution of the *D*_2 − 4_ (μm^2^/s) values of individual PR trajectories exposed to increasing R5020 concentrations for 1 h. Ethanol corresponds to the control condition, i.e., in the absence of the ligand. The *y* axis corresponds to the frequency of events. Vertical dash lines indicate *D*_2 − 4_ values 0.0061 (left line) and 0.5 μm^2^/s (right line). Data extracted from at least 1,000 trajectories belonging to at least eight cells from three independent experiments. (*D*) Polar histograms of the angle between successive steps of diffusing PR under increasing R5020 concentrations. (*E*) Anisotropy values as a function of R5020 concentration for at least eight cells analyzed. Results of a one-way ANOVA test are shown as n.s. for not significant, ***P value< 0.001.

As with most nuclear receptors, PR contains an intrinsically disordered N-terminal domain region (*SI Appendix*, Fig. S1) and is thus prone to phase-separate. We first confirmed the LLPS of PR in the nucleus of living breast cancer cells after hormone exposure using confocal microscopy. Condensates visibly formed minutes after adding the hormone (Video S1). In addition, we also tested the LLPS behavior of PR condensates by adding 5% 1,6-hexanediol, a treatment known to dissolve liquid–liquid assemblies (30), and observed that PR condensates readily dissolved after exposure to the alcohol (*SI Appendix*, Fig. S2), consistent with an LLPS process. However, and contrary to a vast literature in the field, PR condensates remained relatively small in size, being clearly diffraction-limited. We thus turned to single-molecule approaches to effectively increase the spatial (∼20 nm) and temporal (∼15 ms) resolution providing dynamic information on the behavior of individual PR molecules in the nucleus. In particular, we applied single-particle tracking (SPT), which has been widely used over the last decade to evaluate the lateral mobility of several TFs and DNA binding proteins in the nucleus of living cells at the single-molecule level (16, 31–35). We generated a stable MCF7 breast cancer cell line expressing a SNAP-GFP-PRB (SNAP (trademark), green fluorescent protein (GFP) and Progesterone Receptor Isoform B (PRB)) (36). PR molecules were labeled with SNAP-JaneliaFluor 549 (JF549) dye (37), and their diffusion inside the nucleus of living cells was recorded under highly inclined illumination at a frame rate of 15 ms, as schematically illustrated in Fig. 1*A*. Individual JF549 localizations were reconnected to generate trajectories that were analyzed by computing the time-averaged mean square displacement (tMSD) and the angular distribution over consecutive steps as shown in Fig. 1*B* and *SI Appendix*, Fig. S3 (32, 33). The instantaneous diffusion coefficients for each trajectory were extracted by linear fitting of the second to fourth points (*D*_2 − 4_) of the tMSD curve (38) and used to build up *D*_2 − 4_ histograms of hundreds of trajectories over different cells (Fig. 1*B*). In addition, the angular distribution provides information on the type of diffusion exhibited by a molecule while interacting with its environment. Whereas the angular distribution is uniform when molecules diffuse in a homogeneous environment, an asymmetric angular distribution with a preferred occurrence of angles at 180° reflects obstacles to the molecule diffusion, the presence of confinement, or diffusion in a viscoelastic environment (29, 33).

To investigate the PR lateral mobility in response to hormones, Michigan Cancer Foundation-7 (MCF7) (Comsa et al., Anti Cancer Research 2015) cells were treated with a broad range of concentrations of the progesterone derivative R5020 (10^−12^ M to 10^−8^ M, for 1 h) or with vehicle (ethanol) as a control (39). As shown in Fig. 1*C*, we mainly observed two populations in the distribution of *D*_2 − 4_ values across different concentrations, similar to other proteins that interact with chromatin (31, 32). Strikingly, instead of a gradual increase in the bound fraction of PRs that one would expect from a stochiometric occupancy of TFs to DNA binding sites with increasing ligand concentration, we found a sharp transition from free to bound fraction taking place at a critical ligand concentration of 10^−10^ M (Fig. 1*C*). This sharp transition in PR mobility suggests that LLPS may be regulating the interaction between PR and chromatin.

We further computed the distribution of angles between consecutive displacements for each individual trajectory on multiple cells and for different hormone concentrations. At hormone concentrations 10^−10^ M and below, diffusion was mainly isotropic and PR explored all angles with equal probability (Fig. 1*D*). In strong contrast, above the critical concentration of 10^−10^ M, the angle distributions became highly anisotropic with an increased occurrence of angles at 180°, i.e., a higher probability for PR molecules to bounce back to their prior position (Fig. 1*D*). To better quantify these results, we computed the degree of anisotropy as the fold increase of angles occurring at 180° ± 30° with respect to 0° ± 30° (32). A sharp transition in anisotropy was retrieved above a 10^−10^ M R5020 concentration (Fig. 1*E*), like that at which the *D*_2 − 4_ sharp transition took place. We interpreted this preferential backward movement as evidence of confinement and an indication of the bias in angles experienced by a particle inside a condensate when being constrained by the condensate boundaries. Altogether, our SPT results were consistent with a ligand-tunable and regulated LLPS process.

## Diffusion Behavior of Individual PR Determined With Machine Learning

Due to the short length of the SPT trajectories (usually less than 30 time segments; *SI Appendix*, Fig. S3), it is challenging to identify the diffusion behavior of PR inside living nuclei using conventional data analysis methods. We thus relied on a recently developed machine learning (ML) analysis (40). Using a combination of convolutional and recurrent neural networks (see *Materials and Methods* and *SI Appendix*, Fig. S4), we 1) identified the theoretical model that best describes the diffusion behavior of individual PR trajectories and 2) determined the corresponding anomalous exponent α, defined as the scaling factor when fitting the tMSD to a power-law ∼ *t^α^*. Here, α = 1 corresponds to Brownian diffusion, α < 1 to anomalous subdiffusion, and α > 1 to superdiffusion. A detailed description of the ML models and their associated errors are presented in *Materials and Methods* and *SI Appendix*, Figs. S4 and S5.

**Fig. 2.**
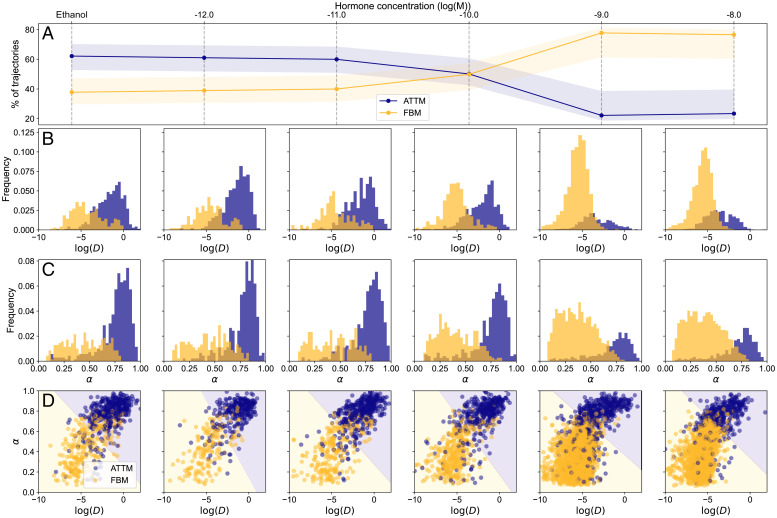
ML analysis of individual PR trajectories in living cells. (*A*) Percentage of trajectories associated to ATTM (blue) or FBM (yellow) by the ML algorithm as a function of ligand concentration. The shadowed areas represent the error of the prediction, calculated by means of a confusion matrix (see *Materials and Methods*) (*B*) *D*_2 − 4_ (μm^2^/s) distributions for varying ligand concentrations, with trajectories associated to ATTM (blue) and FBM (yellow), as identified by ML. (*C*) Corresponding histograms of the ML predicted anomalous exponents. (*D*) Scatter plot of the *D*_2 − 4_ vs. anomalous exponent for every trajectory. Background color represents the prediction of an SVM trained on the data (see *Materials and Methods*).

We first trained the algorithm with a set of simulated trajectories arising from various diffusion models related to many different experimental observations (see *Materials and Methods*). Remarkably, when applied to our single-molecule experimental data, the ML algorithm revealed two main types of diffusion, i.e., the majority of the trajectories were classified as either diffusing according to the annealed transit time model (ATTM) (41) or exhibiting fractional Brownian motion (FBM) (42). The ATTM has been associated with the anomalous, nonergodic, and non-Gaussian motion of particles diffusing in a spatiotemporal heterogeneous medium, e.g., on cell membranes (43). More precisely, the ATTM considers that particles diffuse in a Brownian fashion but experience random changes of diffusion coefficients due to their inhomogeneous environment, resulting in anomalous diffusion behavior (α < 1). FBM has been described as an extension of Brownian motion where the motion of the particle exhibits correlated noise and correlated displacements. In the case of negatively correlated displacements, the diffusion is anomalous (α < 1). Such negative correlations have often been used to describe the motion of particles in viscoelastic media such as the cytoplasm and nucleoplasm of living cells (44, 45) (see *SI Appendix*, Note S1 and Fig. S6 for more details of both models). Note that since the trajectories were normalized before entering the ML architecture (see *Materials and Methods*), the ML prediction was independent of the diffusion coefficient value. For each hormone concentration, we computed the percentage of trajectories predicted as ATTM or FBM. At ligand concentrations below 10^−10^ M, 60% of the trajectories were classified as ATTM and 40% as exhibiting FBM (Fig. 2*A*). Notably, a sharp change in the diffusion behavior occurred at > 10^−10^ M R5020, with ∼80% of the trajectories exhibiting FBM and ∼20% exhibiting ATTM (Fig. 2*A*). We further exploited the powerful discrimination capability of the ML algorithm to compute the *D*_2 − 4_ values of the trajectories assigned to each of the theoretical models. We found that FBM trajectories displayed a much lower lateral mobility as compared to those assigned to the ATTM (Fig. 2*B*). Together, the sharp increase in the number of molecules exhibiting FBM and their lower mobility at ligand concentrations above 10^−10^ M suggest that PR diffusion behavior results from viscoelastic interactions between the receptor and chromatin within a condensate.

Using a different ML architecture as described in *Materials and Methods*, we also predicted the α values for each of the observed trajectories. We found that FBM trajectories exhibited on average lower α values (0.43 ± 0.07) than ATTM trajectories (0.72 ± 0.08) (Fig. 2*C*). To assess the relationship between *D* and α, we generated scatterplots of these two parameters for different ligand concentrations (Fig. 2*D*). Strikingly, trajectories assigned to either ATTM or FBM formed two differentiated clusters that could be readily classified using a support vector machine (SVM), a common supervised learning technique, known for its robustness and easy applicability in low-dimensional problems (46). The SVM was trained to predict the diffusion model given the fitted *D* and the ML-predicted *α.* The background color used in Fig. 2*D* shows the predictions of the SVM over the whole range of *D* and α, demonstrating that they were sufficient to separate the lateral diffusion behavior of individual PRs as a function of ligand concentration. Overall, the ML analysis accurately separates two PR populations diffusing in markedly different media; and most important, it reflects a critical ligand concentration at which a transition from unbound (ATTM) to chromatin-bound (FBM) takes place.

## Nanometer-Scale Temporal Evolution of PR Condensates in Living Nuclei

**Fig. 3.**
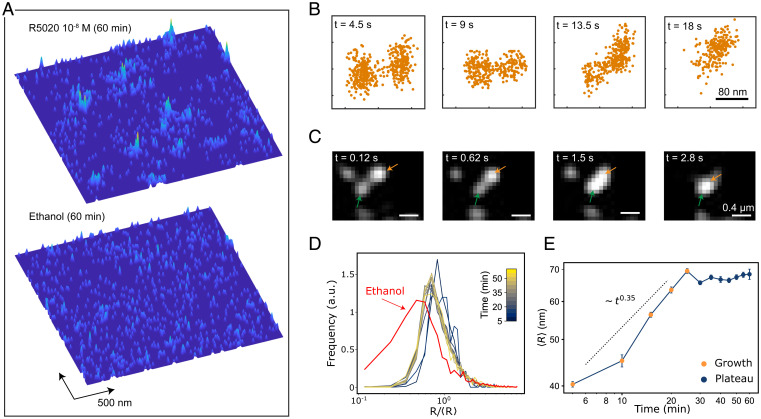
Nanometer–scale spatiotemporal mapping of PR in living nuclei. (*A*) 2D density maps of individual PR localizations collected over 75 s on an area of 2.4 × 2.4 μm^2^, after 1 h ligand stimulation (*Top*) and control (*Bottom*). Each map contains 1,000 localizations. (*B*) Snapshots of two different condensates as they merge over the indicated time windows. The 2D maps have been generated by accumulating single-molecule localizations in time windows of 4.5 s (300 frames). (*C*) Merging events of two different PR condensates (highlighted by orange and green arrows) visualized by confocal microscopy using GFP labeling conditions. (*D*) Distribution of PR condensate radius normalized to the mean radius, over a time course of 60 min after 10^−8^ M hormone stimulation (see color bar). Each curve corresponds to a 5-min time point. The red curve corresponds to the size distribution in the absence of the hormone. a.u. refers to arbitrary units. (*E*) Mean condensate radius as a function of time. At each time point, data correspond to several regions of interest analyzed from two different cells and two separate experiments.

Our single-molecule mobility analysis was consistent with the emergence of PR condensates in living nuclei above a critical ligand concentration, but it did not provide direct information on the condensate sizes. To inquire on the relevant spatiotemporal scales involved in PR condensation and its temporal evolution, we took advantage of the nanometer localization precision encoded in the SPT data. We used this information to generate 2-dimensional (2D) density maps of individual PR localization positions as the receptor dynamically explored the nuclear region (38). The 2D maps clearly showed that hormone treatment (10^−8^ M, 60 min) led to a strong accumulation of single-molecule localization events in small regions, as compared to control conditions (Fig. 3*A*). We further evaluated the lateral mobility inside condensates by reconnecting the localization positions over consecutive frames. Remarkably, PR trajectories within condensates reproduced the mobility, angle distribution, and FBM diffusion behavior of the slow population retrieved by standard SPT shown in Figs. 1 and 2 (*SI Appendix*, Fig. S7). These results indicate that the slow population retrieved from the SPT analysis over all trajectories corresponds to the diffusion of PR molecules *inside* condensates rather than to the diffusion of the condensate itself. Interestingly, when we generated the cumulative probability distribution function of square displacements for these trajectories (see *Materials and Methods*), we retrieved two subpopulations with distinct diffusion coefficients (*SI Appendix*, Fig. S8 *A–C*). A large fraction of the trajectories inside condensates (∼90%) exhibited slow diffusion (< 0.001 μm^2^/s) while ∼10% of the trajectories showed a higher mobility (*D* = 0.04 μm^2^/s, but still slower than molecules outside condensates, i.e., *D* = 0.5 μm^2^/s). These results suggest that *within* condensates, a majority of PR molecules are bound to DNA and thus exhibit a much lower diffusion coefficient. On the other hand, the fraction of PR molecules that diffuse faster within the condensates should then correspond to non-DNA-bound PR (*D* = 0.04 μm^2^/s). Nevertheless, we point out that these data may be somewhat biased toward the occurrence of a higher fraction of slower-diffusing molecules because they stay longer in focus and thus are more prone to being detected, as compared to the faster-diffusing molecules.

To determine the physical mechanism that leads to PR condensation in the nucleus, we first relied on the fact that the 2D density maps also contain temporal information. We thus accumulated localizations for time intervals of 4.5 s (300 frames) to build up the temporal evolution of condensates during an observation time of 18 s and used a cluster algorithm (47) (see *Materials and Methods*) to detect condensates formed by the local accumulation of individual localizations. We readily observed merging events of individual condensates in time (Fig. 3*B*) that were also confirmed by confocal video imaging at a high temporal resolution of fully saturated GFP-labeled PR molecules (Fig. 3*C*). The merging of condensates is a first indication that its growth is dominated by a BMC process. BMC is characterized by the Brownian diffusion of small condensates, which, upon encountering, fuse to each other to form larger condensates (24) (see also *Materials and Methods* and *SI Appendix*, Fig. S9 for an additional description of BMC).

Since PR condensation in our system could be accurately tuned by the time and amount of hormone addition, we exploited this property to further assess the condensate growth mechanism. For this, we generated 2D density maps of single-molecule localizations over a time course of 60 min starting right after adding the hormone (10^−8^ M). We cumulated localizations over 5-min intervals and used the cluster algorithm to generate distributions of condensate radii at each 5 min time point. Interestingly, we found a log-normal distribution of the condensate radii (Fig. 3*D*), similar to that described for systems undergoing LLPS under a BMC mechanism (27). Note that a similar size distribution was also observed in the absence of hormone (ethanol), indicating a pre-existing population of small condensates, in agreement with our SPT data. In addition, we calculated the mean radius size of the condensates over time (Fig. 3*E*). Two distinct regimes could be clearly identified. During the first 30 min, the average radius grew following a power law, <*R*> ∼ *t^β^*, with a fitted β = 0.3505. After 30 min, the system reached a steady-state plateau in which the average size of condensates remained constant. To further confirm this steady-state plateau using a complementary analysis, we generated a cumulative MSD plot of all trajectories inside condensates after 60 min hormone exposure (*SI Appendix*, Fig. S8*D*). The MSD plot exhibited a plateau, which is characteristic for confined diffusion, from which we extracted a confinement size (∼150 nm), in excellent agreement with the results obtained by means of the 2D density maps. The initial growth scaling exponent, the log-normal distribution of the condensates’ radii, and the presence of merging events were all consistent with a BMC-based condensate growth mechanism (48).

Intriguingly, whereas the classical BMC model predicts that condensates grow in time until forming a single droplet (48), our system clearly deviated at longer times from such a prediction, reaching a plateau with condensates sizes of approximately 70 nm in radius (Fig. 3*E*). To understand such a nanoscale-arrested growth, we took a closer look at our SPT data. Despite the short length of the trajectories, we could readily detect the occurrence of escaping events, i.e., particles being able to exit the condensate (*SI Appendix*, Fig. S10), although their statistical quantification was challenging given the short length of the trajectories. Such escaping behavior has been also recently observed on DNA repair condensates in living cells and thoroughly quantified by comparing trajectory length displacements for molecules entering or escaping from condensates (34). These observations suggest that particle escaping could influence PR condensate growth at the steady state in the nucleus.

## Particle Escaping Leads to Nanoscale-Arrested Growth of PR Condensates

**Fig. 4.**
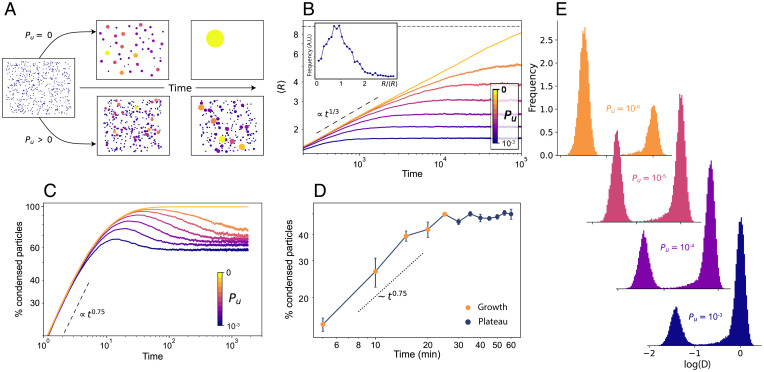
Extended BMC model including stochastic unbinding of PR molecules from condensates. (*A*) Snapshots of two simulations of the theoretical model, showcasing the temporal evolution of two systems, one with *P_u_* = 0 (*Top*) and one with *P_u_* > 0 (*Bottom*). (*B*) Mean radius size evolution as a function of time, for a system of *n* = 80, *L* = √*N*/0.01. Each color represents the result for a different *P_u_*. The dotted line shows the expected BMC growth (<*R*> ∼ *t*^1/3^). The horizontal dashed line shows the maximum mean size possible for the simulated system (<*R*> =√*N*). The inset shows the steady-state normalized radius distribution for a system of *n* = 500 and *L* = √*N*/0.01 for *P_u_* = 0.2, in arbitrary units (a.u.). (*C*) Percentage of particles forming condensates as a function of time for different *P_u_* values. (*D*) Experimental data showing the percentage of particles forming condensates as a function of time. The data correspond to the same experiments shown in Fig. 3 *D* and *E*. (*E*) Diffusion coefficient distributions resulting from the simulations, for free particles (centered around Log(*D*) = 0) and for condensates (left distribution) for four different *P_u_* values. Y axes for all the histograms correspond to Frequency in arbitrary units

To investigate whether the presence of escaping events in a BMC scenario could lead to an arrested growth of condensates with a plateau on their sizes, we developed a theoretical model in which particles—PR dimers in our case, or other biological components in a general context—diffused freely through the system but also interacted with each other in a nontrivial way. The model was based on the main principles of BMC: When two particles coincide (i.e., they contact each other), they interact together, forming a condensate. Subsequent new interactions make the condensates grow until reaching a phase-separated system in which all the particles segregate from the environment, forming a single condensate. To include the effect of particle-escaping events in our model, we simulated a system of particles performing BMC-like condensate growth but incorporating a probability, *P_u_*, that particles escape from condensates (see *Materials and Methods*). Compared to the classical BMC model (Fig. 4*A*, *Top*), the presence of escaping events (i.e., *P_u_* > 0) prevented the system from reaching the single condensate state (Fig. 4*A*, *Bottom*), resembling our experimental observations.

We performed simulations considering that at each time step particles have a probability *P_u_* of unbinding and exiting the droplet in which they are contained, and we calculated the average size of the condensates <*R*> as a function of time (Fig. 4*B* and *SI Appendix*, Fig. S11). We considered that any particle inside a condensate may escape, not only the ones in the condensate surface. Since a large fraction of the PR molecules inside the condensates appear to be bound to DNA, as inferred from their diffusion coefficient (*SI Appendix*, Fig. S8), we hypothesized that such a mechanism corresponds to PR molecules unbinding from their respective DNA binding sites. Nonetheless, the difference between volumetric or surface escaping is negligible for small systems, as well as for the small size of the condensates observed in our experiments (*SI Appendix*, Fig. S12 and Note S2). For BMC (*P_u_* = 0), the system grows following the expected relation <*R*> ∼ *t*^1/3^ with a final single condensate size (horizontal dashed line in Fig. 4*B*). For values of *P_u_* > 0, condensate growth follows the same power law scaling, but notably, the system reaches a plateau with a steady-state mean radius <*R*>_∞_ of a smaller size, akin to our experimental observations. The larger the escaping probability, the smaller the final radius of the condensates (*SI Appendix*, Fig. S12). Using the simulations, we also generated distributions of the steady-state condensate sizes for different *P_u_*. An example of such distribution for *P_u_* = 0.2 is shown in the inset of Fig. 4*B*, exhibiting the expected log-normal distribution for BMC processes. Notably, the steady-state size distribution derived from the simulations was qualitatively similar to that obtained from our experimentally generated 2D density maps (Fig. 3*D*).

To further validate our model in terms of predictions that could be experimentally tested, we calculated from the simulations the percentage of condensed particles as a function of time, for different *P_u_*. For a standard BMC process (*P_u_* = 0), in the steady-state regime a single condensate will be formed, and accordingly, the percentage of condensed particles should reach ∼100%. However, in a scenario in which particles have a certain probability for escaping a condensate, the balance between coalescing and escaping events should maintain the percentage of condensed particles as constant after the initial growth period. Our model predicted that the percentage of condensed particles increases as ∼ *t* ^0.75^ for shorter times and reaches a plateau with a constant percentage of condensed particles, whose value is again dependent on *P_u_* (Fig. 4*C*). To experimentally test this prediction, we extracted the percentage of condensed particles from our experimental 2D density maps at different ligand exposure times (10^−8^ M hormone concentration). Remarkably, our experimental data showed an increase in particle condensation at early growth times with a similar exponent to the one predicted by our model, and most important, it also exhibited a plateau in the percentage of particles forming condensates after 30 min hormone exposure (Fig. 4*D*). Hence, our model of BMC–condensate growth together with condensate particle escaping could fully recapitulate our experimental data and make predictions fully testable at the single-molecule level in living cells.

Finally, we generated diffusion coefficient histograms from our simulations. Since our experimental data were consistent with a standard BMC mechanism, i.e., condensate growth scales with ∼ *t*^1/3^, being valid for Brownian diffusion of condensates (28), we considered in our model the presence of Stokes drag. Hence, free particles (i.e., outside condensates) would diffuse with the diffusion coefficient *D,* while the actual condensates of size *R* would diffuse with the diffusion coefficient *D_r_* = *D*/*R*. Moreover, to account for the heterogeneities present in any biological scenario, we added a small random noise to each *D* value. We generated in silico distributions of the *D* values at steady state for various *P_u_* (i.e., accounting for the final condensate radius *R* and the percentage of free vs. condensed particles at each given *P_u_*). As expected, Fig. 4*E* shows the appearance of two distinct distributions, with a peak at *D* = 1 corresponding to the diffusion of free particles and a second peak at lower *D*, which was an effect of the Stokes drag and hence corresponded to the condensates diffusion. Interestingly, decreasing *P_u_* effectively increased the number and sizes of the condensates and reduced the number of free particles, resulting in a similar effect to the increase of hormone concentration. Based on these results, we propose that at low hormone concentrations, the escaping probability of PR molecules from small condensates is large, leading to a large number of free, noncondensed particles. As hormone concentration increases beyond a critical value, the PR escaping probability reduces so that condensates grow reaching a finite stable size that is ultimately controlled by *P_u_*.

## Discussion

We have presented a single-molecule study of the physical properties of transcriptional condensates in living cells. The inducibility of our system to undergo phase separation by means of hormone concentration and exposure time allowed us to accurately tune the onset of phase separation and thoroughly investigate the growth dynamics of nuclear PR condensates in living cells. Interestingly, we found that while growth dynamics of PR condensates are dominated by BMC at shorter times, condensates exhibited arrested growth, reaching nanoscale sizes at longer timescales, clearly deviating from a classical BMC mechanism. To rationalize our results, we proposed an extension of the BMC model by including the stochastic unbinding of particles within condensates, i.e., introducing a probability that considers particle escaping from condensates. With this minimal consideration, our model fully reproduces the key features of an experimental system undergoing phase separation in living cells. Moreover, by modulating the probability of particle escaping, our model can predict the final condensate sizes and the population of molecules partitioning inside or outside condensates as well as their diffusion behavior. Although in our study we did not pursue further analysis on the occurrence of particle escaping given the short length of the trajectories, recent work has experimentally observed similar escaping from condensates and thoroughly analyzed these events at the single-molecule level (34). Such statistics are not required in our case as our model solely relies on their existence. In fact, a low escaping probability in our model is enough to fully recapitulate the experimental data. Nevertheless, it would be interesting to investigate the statistics of particle escaping and of molecules crossing the condensate boundaries by following the statistics of single-molecule displacements as performed by others (49).

As a whole, our experimental data and theoretical model are consistent with droplet growth dynamics being ultimately controlled by the escaping probability of TF molecules within condensates. The interplay between condensation assembly and single-molecule escaping thus supports a preferred and maximum physical condensate size. Particle escaping from condensates can account for an exquisite control of the condensate size in nonequilibrium systems such as the cell, as also recently observed in other biological scenarios such as DNA repair condensates (34). This mechanism may provide a delicate fine-tuning by the cell that prevents a single phase that would lead to transcription collapse or chromatin condensation.

Recently, some debate has been raised discussing the exact mechanism leading to phase separation in the nucleus of living cells. While most of our experimental data point to an LLPS mechanism, the presence of two distinct PR diffusing populations within the condensates can also be related to the presence of chromatin binding sites. We note that the model presented in this work is agnostic regarding the binding mechanism causing the formation of condensates and can indeed be applied to both LLPS and a polymer–polymer binding model. While differentiating between these two models is beyond the scope of our contribution, recent work has shed light on possible single-molecule observables to distinguish between both (13).

Recent SPT experiments showed that TFs transiently bind to DNA with rather short binding times (in the seconds scale) (16, 17). We propose that condensate formation may increase the likelihood that individual PRs rebind within short timescales to their corresponding DNA binding region. Such a condensate environment will thus increase the effective time that a given DNA region is bound by TFs (50). This hypothesis is further substantiated by our experimental data analyzed by ML, where FBM, traditionally associated with diffusion within viscoelastic media, was found to describe best PR low mobility diffusion. In conclusion, the combination of single-molecule sensitive imaging techniques together with theory and simulations as reported here contributes a substantial step forward in understanding the behavior of individual proteins within condensates.

TF condensation has been customarily studied through ensemble or static measurements, mostly in in vitro settings or in fixed cells. In contrast, the experiments and theoretical model presented here provide a general framework to investigate the dynamics of phase separation in living cells at the single-molecule level. Moreover, our approach can be further extended to a wide range of biological systems as well as other soft-matter–based interacting systems. Overall, this work offers insights into phase separation in soft-matter systems from both experimental and theoretical perspectives.

## Materials and Methods

### Plasmids.

The original pGFP-PRB was a gift from Gordon Hager (National Cancer Institute, NIH, Bethesda, MD). This plasmid expresses the PR isoform B under a tetracycline controllable promoter (TetOff system, Clontech). To perform the SPT experiments, a SNAP tag was introduced at the N-terminal to the GFP, using Gibson cloning (pSNAP-GFP-PRB). A puromycin resistance plasmid (pPUR, Clontech, catalog no. 631601) was used as a selection marker. All plasmids were linearized with ScaI before electroporation.

### Cell culture and electroporation.

MCF7 Tet-off cells (Clontech, catalog no. 631154) were grown on DMEM high-glucose media supplemented with 10% Tet-free FBS, 2mM L-glutamine, 1 mM sodium pyruvate, 100 U/mL-1 penicillin, and 100 μg/mL-1 streptomycin. The cells were cultured at 37 °C in a CO_2_/air (5%/95%) incubator. Cells were electroporated simultaneously with the pSNAP-GFP-PRB and the pPUR, using a 10:1 ratio, respectively. Electroporation was performed using the Amaxa Cell Line Nucleofector Kit V (Lonza) using the P-20 program, following the manufacturer’s instructions. After 1 week, cells were selected under 0.6 μg/mL puromycin to enrich for electroporated cells, and they were then sorted in single-cell wells using GFP as a marker in order to generate a stable cell line.

### Hormone stimulation and SNAP labeling.

Two days before the microscopy, approximately 200,000 cells were seeded in 35-mm glass-bottom dishes. Sixteen hours before hormone stimulation, cells were washed with PBS solution to eliminate traces of phenol red, and then they were changed to white DMEM media supplemented with 10% charcoal-treated FBS serum, 2 mM L-glutamine, 1 mM sodium pyruvate, 100 μg/mL-1 penicillin, and 100 μg/mL-1 streptomycin; this combination is hereafter abbreviated as “charcoalized white DMEM.” The JF549 dye coupled to the SNAP substrate was kindly provided by Luke Lavis (Janelia Farm, Ashburn, VA). Cells were incubated with 10 nM for SPT and 100 nM for 2D spatiotemporal maps of the SNAP JF549 dye in charcoalized white DMEM for 30 min at 37 °C. Subsequently the cells were washed three times with PBS and then placed back in the incubator in charcoalized white DMEM for 1 h washout at 37 °C. After the JF549 SNAP labeling, hormone stimulation was done using R5020 (promegestone) solubilized in ethanol or using control conditions with this solvent. To study the response to different concentrations of hormone, a series of dilutions was made freshly before the microscopy acquisition. Time course experiments were performed at a hormone concentration of 10^−8^ M, and SPT tracking data were recorded at intervals of 5 min during a total observation time of 60 min.

### 1,6-Hexanediol treatment.

To test whether LLPS might be regulating the emergence of PR condensates, we treated R5020-exposed MCF7 cells with 5% 1,6-hexanediol dissolved in R5020 containing cell medium. Using confocal microscopy, we could observe the dissolution of PR condensates already after 5 min 5% 1,6-hexanediol treatment. In order to maintain cell integrity, we did not perform the treatment for longer than 15 min.

### Experimental setups.

SPT and 2D spatiotemporal density maps imaging were performed in a Nikon *N*-STORM 4.0 microscope system for localization-based superresolution microscopy, equipped with a TIRF 100×, 1.49 NA objective (Nikon, CFI SR HP Apochromat TIRF 100XC Oil). The sample was illuminated by a continuous 561 nm laser line with a power of 30 mW before the objective in highly inclined and laminated optical sheet (HILO) configuration. The emission fluorescence of the JF549 dye was collected through the objective and projected into an EM-CCD Andor Ixon Ultra Camera at a frame rate of 15 ms. The pixel size of the camera is 160 nm. During imaging, the temperature was kept at 37 °C in an incubation chamber. GFP confocal line scanning microscopy was performed in a Leica TCS SP5 II CW-STED microscope using a 63× Oil, 1.4 NA objective (Leica HC PL APO 63×/1.40 Oil CS) using a multiline Argon laser at 488 nm for excitation. The emission fluorescence was detected with a Hybrid detector (Leica HyD) in photon counting mode, using a 500 to 550 nm filtering. The sample was kept at 37 °C with 5% CO_2_ by an incubation chamber. For Fig. 3*C* and *SI Appendix*, Fig. S2, images of 256 × 256 pixels were acquired with a pixel size of 80 nm and a dwell time of 9 μs. Scanning was performed at 100 Hz, acquiring consecutive frames every 125 ms. For Video S1, images of 322 × 200 pixels were acquired with a pixel size of 160 nm. Each frame in the video has a total integration time of 15 s and corresponds to the sum intensity projection from 100 images taken consecutively every 150 ms, scanning at 700 Hz.

### Data analysis.

To generate SPT trajectories, the nuclear region was segmented in the GFP channel intensity using Fiji. Individual tracks inside the nuclear region were analyzed using Trackmate (51). Particle detection was performed with a difference of Gaussians, with an expected diameter of 0.6 μm and subpixel localization. Detected particles were first filtered based on the signal-to-noise ratio of the input image and then based on the quality score. The particles retained were then linked using a simple linear assignment problem tracker, with a 1-μm linking distance, a 1-μm gap closing maximum distance, and a gap closing of two frames. Only tracks with more than 10 frames were considered for the analysis. A histogram of the number of frames for the trajectories used in this study at every ligand concentration is presented in *SI Appendix*, Fig. S3*A*.

To generate 2D spatiotemporal maps, the total single-molecule localizations of JF549-labeled PR molecules were detected using custom Matlab Software over 5,000 frames (75 s) and projected into one single frame. Condensates were detected by applying density-based spatial clustering of applications with noise (47) over the entire frame with a threshold of 48 nm of interparticle distance and condensates containing a minimum number of particles of 5. The radius was extracted by considering the area of the condensate a circle. The percentage of free particles was estimated by the number of particles not detected within a cluster divided by the total number of particles within a given area. The escaping-events analysis was performed by taking PR trajectories and detecting within each trajectory a cluster by the clustering algorithm. Only trajectories where there were clear escaping events were considered. Time-evolution 2D density maps were generated by cumulating localization positions every 4.5 s, corresponding to 300 frames, for a total duration of 18 s.

Given a trajectory whose 2D position (*x, y*) was sampled at T discrete, regular time steps *t_i_*, its tMSD was calculated using the following equation (52):

[1] $$\text{tMSD}(\Delta )=\frac{1}{T-\Delta }\sum_{i=1}^{T-\Delta }\left({\left[x\left(t_{i}+\Delta \right)-x\left(t_{i}\right)\right]}^{2}+{\left[y\left(t_{i}+\Delta \right)-y\left(t_{i}\right)\right]}^{2}\right),$$

where Δ is usually referred to as the time lag. Even in the presence of anomalous diffusion, at short times the MSD was well represented by

[2] tMSD=4DΔ+offset,

where *D* is the instantaneous diffusion coefficient. To extract *D*, we fit the tMSD between Δ = 2 and Δ = 4 and redefined it, as presented in the main text, as *D*_2 − 4_. Examples of tMSD for every ligand concentration are presented in *SI Appendix*, Fig. S3 *B–G*.

To extract the size of the condensates, we generated a cumulative tMSD plot from all segments of the trajectories inside the condensates (shown in *SI Appendix*, Fig. S8*D*). The cumulative plot was fitted with the following equation (53):

[3] $$r_{i}^{2}\left(t\right)=a+\left(\frac{L_{i}^{2}}{3}\right)\left(1-e^{\left(\frac{\Delta t}{t_{i}}\right)}\right),$$

where *a*, *L*, and *t* are the offset, the size of the confinement, and the average duration, respectively.

The cumulative probability distribution function of squared displacements at different time lags was fitted with a two-component Fick’s law–based function (54):

[4] $$P\left(r^{2},t\right)=1-\left[s*e^{\frac{r^{2}}{r_{s}^{2}}}+\left(1-s\right)*e^{\left(-\frac{r^{2}}{r_{f}^{2}}\right)}\right],$$

where *P(r^2^,t)* is the probability that a particle is diffused within a circle with radius *r* at a given time lag *t*. The fitting yielded the fraction of slowly diffusing molecules and the tMSD of the slow and fast fractions, which was fitted with:

[5] tMSD=4DΔ+offset,

to obtain the *D_2–4_* of each fraction.

For a given time, *t*, and a time between frames, δt, we defined the turning angle, θ*_t_*, between consecutive trajectory segments, $\vec{s}\left(t,\;t+\;\delta t\right)=\;\vec{r}\;\left(t+\delta t\right)-\vec{r}\left(t\right),\;$ as follows (55):

[6] $$\theta_{t}=\tan^{-1}\left(\frac{\vec{s}\left(t,t+\delta t\right)\times \vec{s}\;\left(t+\delta t,t+2\delta t\right)}{\vec{s}\left(t,t+\delta t\right)\cdot \vec{s}\left(t+\delta t,t+2\delta t\right)}\right).$$

For our calculations, we considered the particle positions to be in three dimensions with the *z* component equal to zero. Using the above expression, the angles are defined between 0° and 360°. To calculate the anisotropy of the turning angles, the fold change between the number of angles from 180° ± 30° and 0° ± 30° was extracted (32). A step-by-step implementation of these calculations is presented in the public repository accompanying this work (see Data Availability).

### ML architecture and analysis.

A schematic pipeline of the ML method used in this study is presented in *SI Appendix*, Fig. S4. The ML architecture is trained with a set of simulated trajectories, generated via the ANDI-datasets Python package (56). This tool allows one to generate trajectories that are assigned to five different diffusion models. Moreover, trajectories with different anomalous exponents (0 < α < 1) can also be generated. The ML architecture can be trained separately to perform two different tasks: 1) to classify the trajectories among a pool of different theoretical models, and 2) to regress the value of the anomalous exponent of each trajectory. Notably, the training is done in a supervised way, i.e., we feed the trajectories to the machine, together with their corresponding labels (either the diffusion models for the first task, or the exponents for the second). A detailed description of the use of ML for characterizing anomalous diffusion trajectories can be found in the public repository accompanying this work (see Data Availability). Moreover, more details on this approach can be found in reference (56) for a Python version and in reference (57, 58) for a Matlab version.  In this paper, we use as architecture a combination of gated recurrent units (GRUs) and convolutional neuronal networks (CNNs) merged with a contact layer made of fully connected neurons as depicted schematically in *SI Appendix*, Fig. S4. The GRU layers can learn long-term features, while the CNNs are a good strategy to tackle short-length correlations (59). By combining the two approaches, we are able to characterize trajectories of only 10 data points in a robust manner.

In order to classify the experimental trajectories according to a given diffusion model, the last layer of the network consists of K neurons, where K is the number of models considered. A softmax function is applied to this last layer. The labels are encoded in a vector of elements, all equal to zero except the one encoding the model of the trajectory. The cost function to minimize is the Kullback–Leibler divergence, which, for a set of trajectories $X=\left\{\vec{x_{1}},\vec{\;x_{2}},\ldots \vec{x_{i}}\right\},$ compares the output vector of the machine $f_{m}(x_{i})$ to the label vector$\;{\vec{y}}_{m}^{\left(i\right)}$ using

[7] $$KL=\sum_{i}^{n}f_{m}\left(x_{i}\right)\log\left(\frac{\;{\vec{y}}_{m}^{\left(i\right)}}{f_{m}\left(x_{i}\right)}\right).$$

To faithfully characterize the set of experimental trajectories, we first trained a model to classify among four diffusion models: continuous-time random walk (60), FBM (42), ATTM (41), and the scaled Brownian motion (61). For each model, we generated trajectories with the anomalous exponent α∈[0.05, 1] in intervals of 0.05. We created a balanced dataset with 1,000 trajectories per model and exponent, which in total summed up to 72,000 trajectories. We separated the dataset into two: a training set with 57,600 trajectories and a test set with 14,400 trajectories. The latter was used to calculate the accuracy of the model, i.e., to prevent the appearance of overfitting. Note that the input size of the machine was fixed, which means that all the input trajectories should have the same size. Because the experimental dataset had trajectories of varying size, from 10 to 1,000 points, we solved such a problem by restricting them to 20 frames long. This procedure ensured that most of the trajectories were considered, while the length was sufficiently large for the machine to have good accuracy. The accuracy was calculated by means of the F1-score, i.e. the harmonic mean between the precision and recall of the model, averaged over all classes globally (often referred to as the microaverage). The trained model then had a microaveraged F1-score of 0.733. When applied to the experimental dataset, 90% of the trajectories were classified as either FBM or ATTM.

Since the vast majority of the trajectories were classified as either FBM or ATTM, we trained the machine only with these two models. This allowed us to increase the accuracy of the ML classification for 20-frame long trajectories. In this case, the F1-score attained was 0.822 (compared to 0.733). The confusion matrix for this classification is shown in *SI Appendix*, Fig. S5*A*. The results of the prediction on the experimental dataset are presented in the main text.

For the anomalous exponent prediction, the output of the machine is a continuous value. Hence, the last layer of the neural network is a single neuron with a rectifier activation function (RELU). The loss function in this problem is the mean absolute error (MAE),

[8] $$\mathrm{MAE}=\sum_{i=1}^{N}\left|y_{i}-f_{e}\left(x_{i}\right)\right|,$$

where $y_{i}$ is the label corresponding the trajectory $x_{i}$, $f_{e}(x_{i})$ is the network prediction, and N is the total number of trajectories in the dataset. The sum is done over the set of trajectories in the training dataset. In order to infer the anomalous exponent for each individual trajectory, we used a simpler version of the neural network, containing two GRU layers of 100 and 50 neurons each whose output entered two fully connected layers of 64 neurons and sigmoid activation functions. The last layer contained a single neuron with a RELU activation function. Between each fully connected layer, we proceeded with a 25% dropout.

To calculate the prediction error, we simulated 10^4^ ATTM and FBM trajectories of length 20, with anomalous exponents ranging from 0.1 to 1. The network showed an MAE of 0.232 in the full dataset. When considering only FBM trajectories, the MAE dropped to 0.158. Moreover, the error distribution was symmetric, as shown in *SI Appendix*, Fig. S5*B*, showcasing that the model was unbiased with reference to the under- or overestimation of the exponent. On the contrary, the model overestimated the exponent when dealing with ATTM trajectories. This was primarily caused by the short length of the trajectories and the characteristics of ATTM. As explained, ATTM models the walk of a particle with random changes of the diffusion coefficient. The distribution of such changes gives rise to anomalous diffusion and is directly related to the anomalous exponent. When dealing with short trajectories, the number of changes is very low or even nonexistent, making it impossible to distinguish such a trajectory to a Brownian motion trajectory. In this kind of trajectory, the model predicts an exponent of 1 or close to 1. Such a feature has to be taken into account when analyzing short ATTM trajectories and finding values close to 1. Nonetheless, we note that such an error regarding the estimation of the anomalous exponent has no implication for the classification of the particles as FBM and ATTM (Fig. 2 *A* and *B*), which is the main result of our ML analysis.

### Theoretical model and simulations.

Our theoretical model is based on the main principles of BMC but with the addition of the stochastic unbinding of particles from already formed condensates. In our system, particles diffuse performing Brownian motion through the system, but they also interact with each other in a nontrivial way. When two particles coincide (i.e., they contact each other), they interact together, forming a condensate. For a standard BMC process, subsequent new interactions make the condensates grow until reaching a phase-separated system in which all the particles segregate from the environment, forming a single condensate. See *SI Appendix*, Fig. S9 for a schematic representation of such a process. However, in our case and motivated by our experimental observations, we include an unbinding probability *P_u_* such that at any given time, particles can exit the condensate in which they are contained.

We performed simulations considering that at each time step, particles have a probability *P_u_* of unbinding and escaping the droplet. As stated in the main text, we assume a uniform escaping mechanism from the condensate that can be attributed to PR molecules unbinding their corresponding DNA binding sites.

The simulations consider the following free parameters:

***N*:** total number of particles;
***r:*** effective radius of the particles. We consider that all particles in the system have the same effective size and that they have a circular shape. If two particles of size *r* are closer than a distance 2*r*, then they coalesce. When the two particles coalesce, the total area is conserved such that the resulting droplet has area 2π*r*^2^. Then, a droplet containing *M* particles has a total radius of $R_{M}=\sqrt{M}r$ and area $A_{M}=M\pi r^{2}$.
***L:*** length of the 2D squared box acting as an environment. The area of the box is hence L^2^. We consider in this case periodic boundary conditions—i.e., any particle traversing one of the borders of the box is immediately transferred to the opposite side. Similar simulations were performed with reflecting boundary conditions with analogous results.
***D:*** Diffusion coefficient of single particles. All particles (free particles and the condensate particles themselves) perform Brownian motion with the same diffusion coefficient *D*. Justified by the experimental results, as well as the theoretical considerations of BMC, we consider the presence of Stokes drag—i.e., a droplet of radius *R* will decrease its diffusion coefficient following *D_R_* = *D/R*.
***P_u:_*** Unbinding probability. At each time step, particles have a probability *P_u_* of unbinding from the droplet in which they are contained. We consider that any particle in the condensate can escape with equal probability. See also *SI Appendix*, Note S2 and Fig. S12 for more details.
***T:*** total number of time steps of the simulation.

The legend for Fig. 4 contains the specific values of the parameters used for the simulations presented.

For simplicity, we usually consider *r* = 1 and *D* = 1. At the start of each simulation, all particles are distributed randomly, following a uniform distribution, all over the environment. The simulation then works as follows:

1. At the beginning of each time step, for every droplet containing more than one particle, we check how many particles unbind. Each particle has a probability *P_u_* of escaping from the droplet it is contained in. All particles that have unbound will not be able to bind until the next time step (i.e., they will not be considered in step 3 below).
2. Each particle or droplet performs a spatial step, sampled from a Gaussian distribution of variance $\sqrt{2D_{r}}$, which effectively samples the steps of a Brownian particle with diffusion coefficient *D_r_*.
3. We iterate over each particle and droplet and find those that are in contact. These are considered to coalesce, forming larger droplets. We consider that the center of the resulting droplet is at the center of mass of the coalescing particles and droplets.
4. Repeat until doing T time steps.

## Supplementary Material

Supplementary File

Supplementary File

## Data Availability

All study data are included in the article and/or supporting information. A representative set of the experimental data used in this paper, as well as the necessary tools to reproduce the results presented in this work can be found in the public repository https://github.com/gorkamunoz/stochastic_unbinding_droplets. A more detailed version of the use of ML for characterizing anomalous diffusion trajectories can be found in reference (56) and in reference (58) for a Matlab version.
