# Supplementary material for: Stochastic particle unbinding modulates growth dynamics and size of transcription factor condensates in living cells
Source: Proc Natl Acad Sci U S A. 2022 Jul 26;119(31):e2200667119. doi: 10.1073/pnas.2200667119 (PMC9351496; doi:10.1073/pnas.2200667119)
Supplement: Supplementary File [file pnas.2200667119.sapp.pdf]

Supplementary information

Stochastic particle unbinding modulates growth dynamics and nanoscale-arrested growth of transcription factor condensates in living cells

Gorka Muñoz-Gil,[1,2] Catalina Romero-Aristizabal,[3] Nicolas Mateos,[1] Felix Campelo,[1] Lara I. de Llobet Cucalon,[3] Miguel Beato,[3,4] Maciej Lewenstein,[1,5] Maria F. Garcia-Parajo,[*1,5] Juan A. Torreno-Pina[*1,3]

[1] *ICFO-Institut de Ciencies Fotoniques, The Barcelona Institute for Science and Technology (BIST), 08860 Barcelona, Spain.*

[2] *Institute for Theoretical Physics, University of Innsbruck, Technikerstr. 21a, A-6020 Innsbruck, Austria*

[3] *Centre de Regulació Genomica (CRG), The Barcelona Institute of Science and Technology (BIST), Dr. Aiguader 88, 08030 Barcelona, Spain.*

[4] *Universitat Pompeu Fabra (UPF), 08020 Barcelona, Spain*

[5] *ICREA, Pg. Lluis Companys 23, 08010 Barcelona, Spain*

[*]***Corresponding authors:***

Maria F. Garcia-Parajo. **E-mail:** maria.garcia-parajo@icfo.eu

Juan A. Torreno-Pina. **E-mail:** Juan.Torreno@icfo.eu

Supplementary Notes

Note S1: Anomalous diffusion models. Below we describe in general the diffusion models considered in this work, namely the annealed transit time model (ATTM) (1) and fractional Brownian motion (FBM) (2). Detailed descriptions of both models can be found in (3, 4).

ATTM considers the motion of a particle with stochastic changes in the diffusion coefficient, as shown schematically in Fig. S6A. In particular, in this work we consider the case in which a particle diffuses for a time τ_1 with diffusion coefficient D_1. After such time, a new tuple (D_2, τ_2) is sampled. Ref. (1) shows that, if the diffusion coefficient follows a power law distribution $P(D) \sim D^{\sigma-1}$, the associated times are calculated via $t = D^{-\gamma}$, and under the condition that $\sigma < \gamma < \sigma + 1$, the resulting trajectories will diffuse with an associated mean squared displacement $\langle x^2(t) \rangle \sim t^\alpha$, with the anomalous exponent $\alpha = \sigma/\gamma$. Examples of ATTM trajectories with different α are shown in Fig. S6A). The resulting motion shows interesting properties, such as weak ergodicity breaking, and has been used to model the heterogeneous diffusion of particles in various physical systems, including the cell membrane of living cells (5).

Fractional Brownian motion (FBM) describes the motion of a particle with correlated displacements (see Fig. S6B). A common representation of such walk is given by means of the Langevin equation

$$\frac{dx(t)}{dt} = \zeta_{fGn}(t),$$

where $\zeta_{fGn}(t)$ is a fractional Gaussian noise. Oppositely to Brownian motion, for which the noise in the equation above is uncorrelated, in this case it follows a power law correlation,

$$\langle \zeta_{fGn}(t_1)\zeta_{fGn}(t_2) \rangle = \alpha(\alpha - 1)K_\alpha^* |t_1 - t_2|^{\alpha-2},$$

where K_α^* is defined as the effective diffusion coefficient. Importantly, the walk arising from the two equations above also shows a mean square displacement $\langle x^2(t) \rangle \sim t^\alpha$. However, and as opposed to ATTM, which is a strictly subdiffusive walk, FBM can have any $\alpha \in [0,2]$. A special property of FBM is that its displacements can be either positively (for $\alpha > 1$) or negatively correlated (for $\alpha < 1$). Examples of FBM trajectories with different α are presented in Fig. S6B. In the case of subdiffusive motion, such negative

correlations have been often used to describe the motion of a particle in viscoelastic media (3).

In summary, both ATTM and FBM models can display anomalous diffusion exponents $\alpha < 1$. However, the physical mechanisms leading to such anomalous behavior are of different origin. In our case, we found that the diffusion of PR molecules inside the nucleus but outside condensates is better described by the ATTM model, whereas PR diffusion inside condensates follow FBM.

Note S2. Volumetric versus surface effects in condensate growth. In our theoretical model, we consider that any particle in the condensate can escape with equal probability. It is important to note that, in some scenarios, only particles in the surface may be able to escape from a condensate. This causes that, given a condensate of size *N*, only $\sim\sqrt{N}$ particles are able to escape (this is only valid in cases in which $\sqrt{N} < N$). As less particles escape from a condensate, systems tend to show less growth arrest. Indeed, previous works have shown that a volumetric term is needed to suppress the growth of a system showcasing Ostwald ripening (6). This may be true in our case, but only when approaching the thermodynamic limit. Also, as the unbinding probability increases, the maximum size attained by condensates may fall below the threshold in which $\sqrt{N} < N$, hence escaping from the surface is similar as being able to escape from any point within the condensate. As we decrease P_u in our model, this effect enters into play, even restricting the appearance of the characteristic plateau in the condensate mean size showcased by our model. Such behavior for large values of P_u is illustrated in Fig. S12.

References:

1. P. Massignan *et al.*, Nonergodic subdiffusion from Brownian motion in an inhomogeneous medium. *Phys Rev Lett* **112**, 150603 (2014).
2. B. B. Mandelbrot, J. W. Van Ness, Fractional brownian motions, fractional noises and applications. *SIAM Rev* **10**, 422-437 (1968).
3. R. Metzler, J. H. Jeon, A. G. Cherstvy, E. Barkai, Anomalous diffusion models and their properties: non-stationarity, non-ergodicity, and ageing at the centenary of single particle tracking. *Phys Chem Chem Phys* **16**, 24128-24164 (2014).
4. G. Munoz-Gil *et al.*, Objective comparison of methods to decode anomalous diffusion. *Nat Commun* **12**, 6253 (2021).

5. C. Manzo *et al.*, Weak ergodicity breaking of receptor motion in living cells stemming from random diffusivity. *Phys Rev X* **5**, 011021 (2015).
6. D. Zwicker, A. A. Hyman, F. Julicher, Suppression of Ostwald ripening in active emulsions. *Phys Rev E Stat Nonlin Soft Matter Phys* **92**, 012317 (2015).

Video S1. Time lapse of inducible PR nuclear condensates. MCF7 cell-line expressing GFP-PRB before and after hormone stimulation. Before treatment with hormone the fluorescent signal of the GFP-PRB is homogeneous across the nucleoplasm. After hormone addition (R5020, 10^{-8} M, black frames) the fluorescent signal distributes into condensates within 5 minutes of hormone exposure. Each frame has a total integration time of 15 s (see Methods for details regarding imaging conditions).

Supplementary Figures:

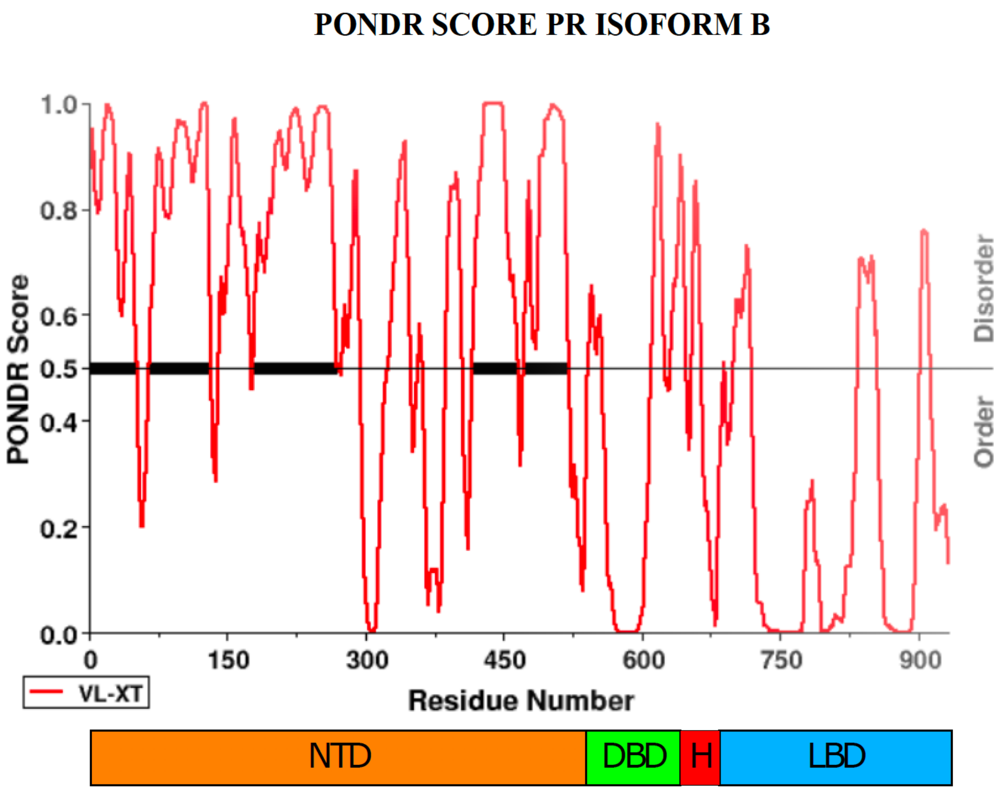

Figure S1. PONDR score of PRB. Prediction of Natural Disordered Regions (PONDR score) of PR-B generated at www.pondr.com. Note the different regions of PR-B denoted as N-terminal domain (NTD), DNA-binding domain (DBD), the Hinge (H) and the ligand binding domain (LBD). The NTD is highly disordered (PONDR score > 0.5).

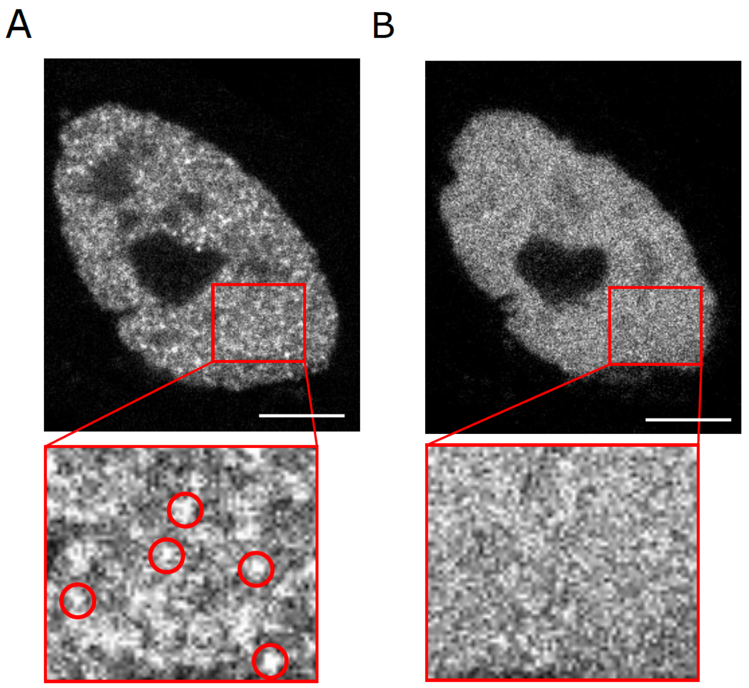

Figure S2. 5% 1,6-Hexanediol dissolves PR nanoclusters in breast cancer cells exposed to hormone. (A) A breast cancer cell was exposed to 10 nM R5020 leading to the formation of PR condensates that can be identified as a punctuated pattern in the entire nucleus. PR condensates are highlighted as red circles in the zoom-in region. (B) Adding 5% 1,6-Hexanediol completely dissolves the PR condensates leading to a homogenous fluorescence signal pattern. Scale bar: 5 µm.

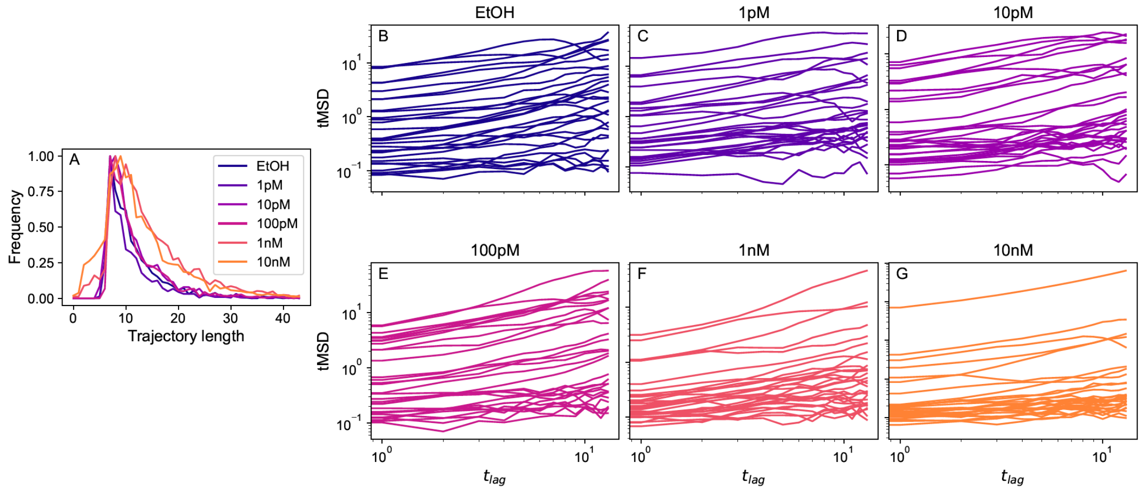

Figure S3. Experimental SPT trajectories. (A) Histogram of the trajectory lengths from the trajectories analyzed for the different ligand concentrations. (B-G) tMSD curves of 30 representative trajectories for each ligand concentration.

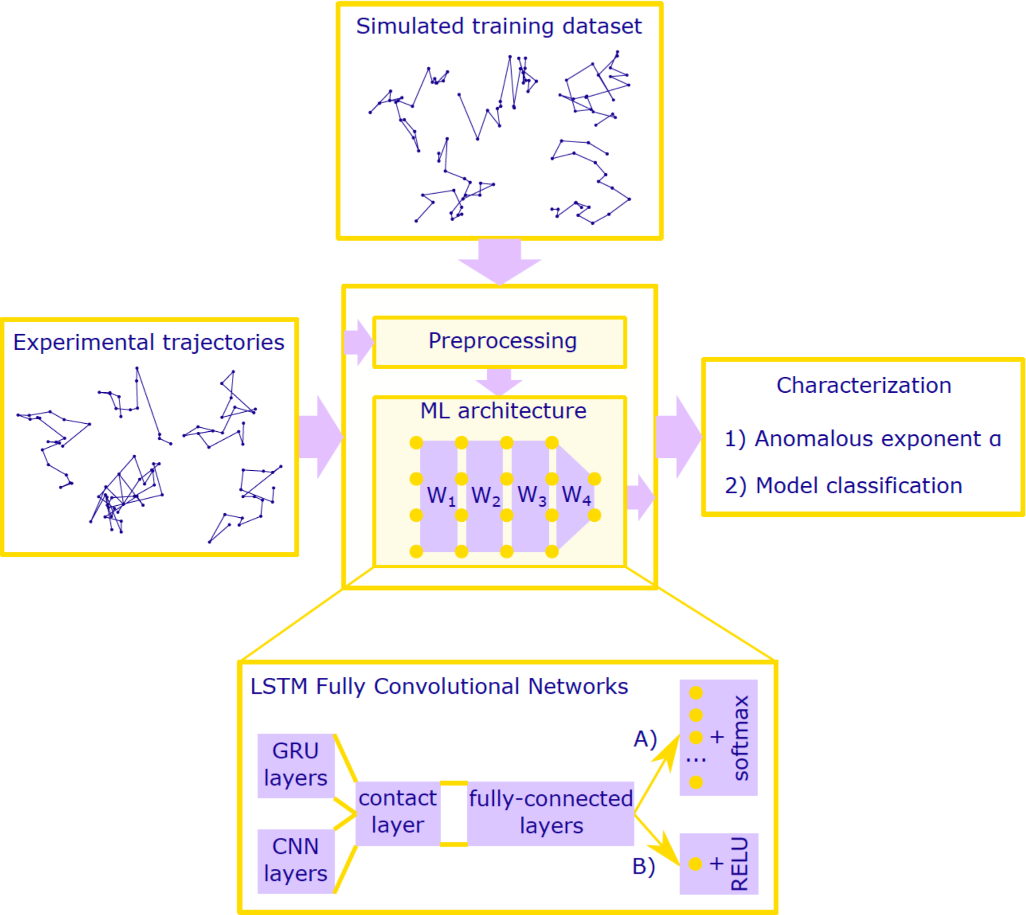

Figure S4. Scheme of the machine learning procedure. The machine learning (ML) architecture is trained with a dataset consisting of simulated trajectories. Once the training is complete, the machine can assign to every experimental trajectory an anomalous exponent and a diffusion model.

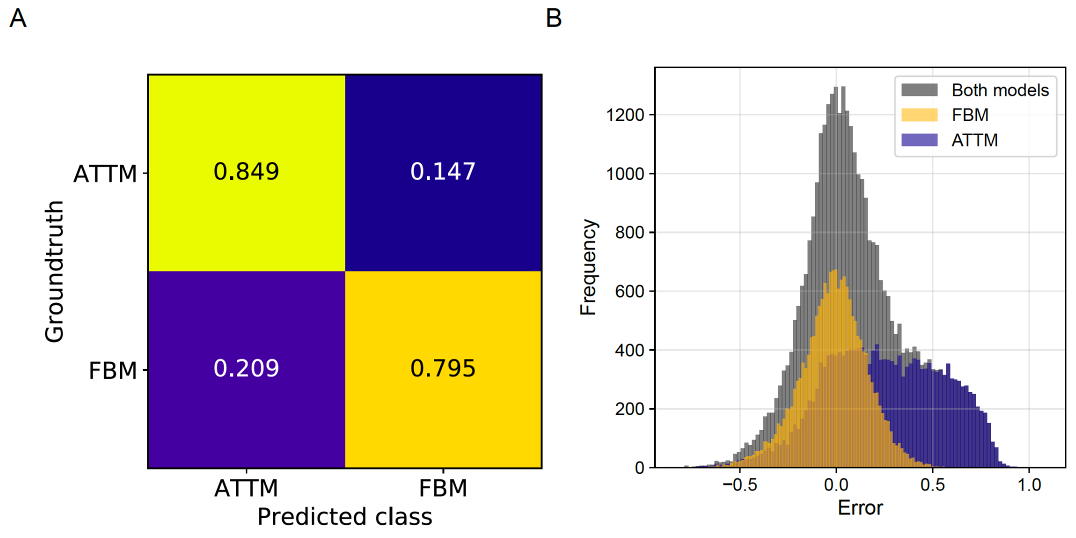

Figure S5. Error in the ML analysis. (A) Confusion matrix for the LSTM Fully convolutional network used for model classification. (B) Prediction error, defined here as $\varepsilon = \alpha_{pred} - \alpha_{true}$, for the GRU network used in the anomalous exponent prediction. In grey we present the error distribution when considering trajectories of both models (MAE = 0.228), orange and blue present the error for trajectories of FBM (MAE = 0.135) and ATTM (MAE = 0.321), respectively. For both cases, results were obtained using 18000 trajectories with trajectory length T = 20, never seen by the machine, in order to avoid any overfitting.

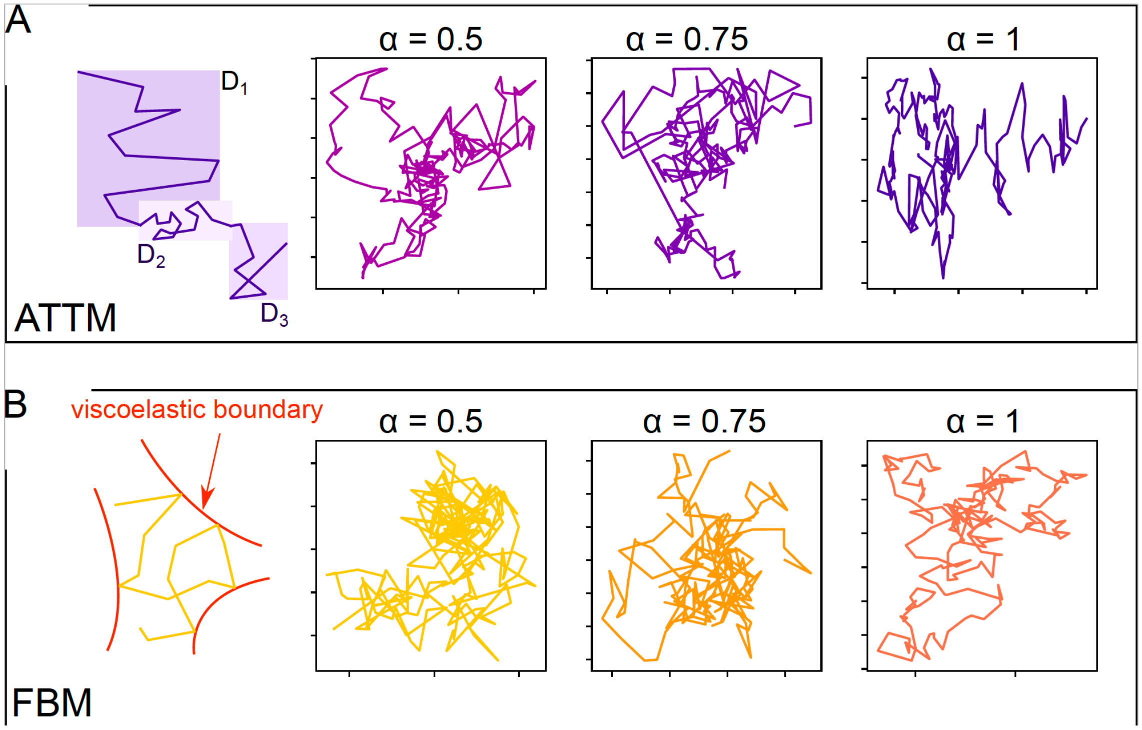

Figure S6: Schematic representation of diffusion models. (A-B) Leftmost panel: Idealized representation of a trajectory exhibiting ATTM (A) or FBM (B) diffusion. Under an ATTM scenario, the particle undergoes random changes in diffusion coefficient

as its explores different environments (highlighted as squared purpled regions of different grading). In FBM, the diffusion of the particle is usually related to the viscoelastic interaction with its environment. Right panels: examples of trajectories with different anomalous exponents. Notice that both models can render similar anomalous exponents, nevertheless their physical origin are different.

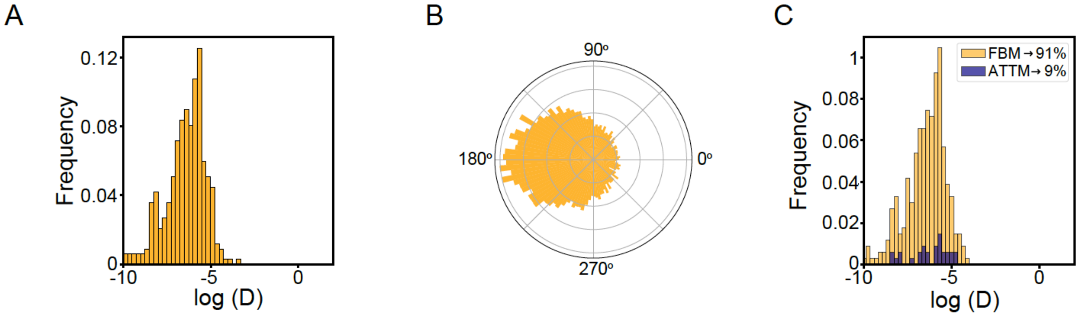

Figure S7. 2D density maps confirms SPT data. (A) Distribution of the $D_{2\text{-}4}$ values of PR trajectories inside condensates. (B) Corresponding angle distribution between successive steps, and (C) ML trajectory assignment to the diffusion behavior.

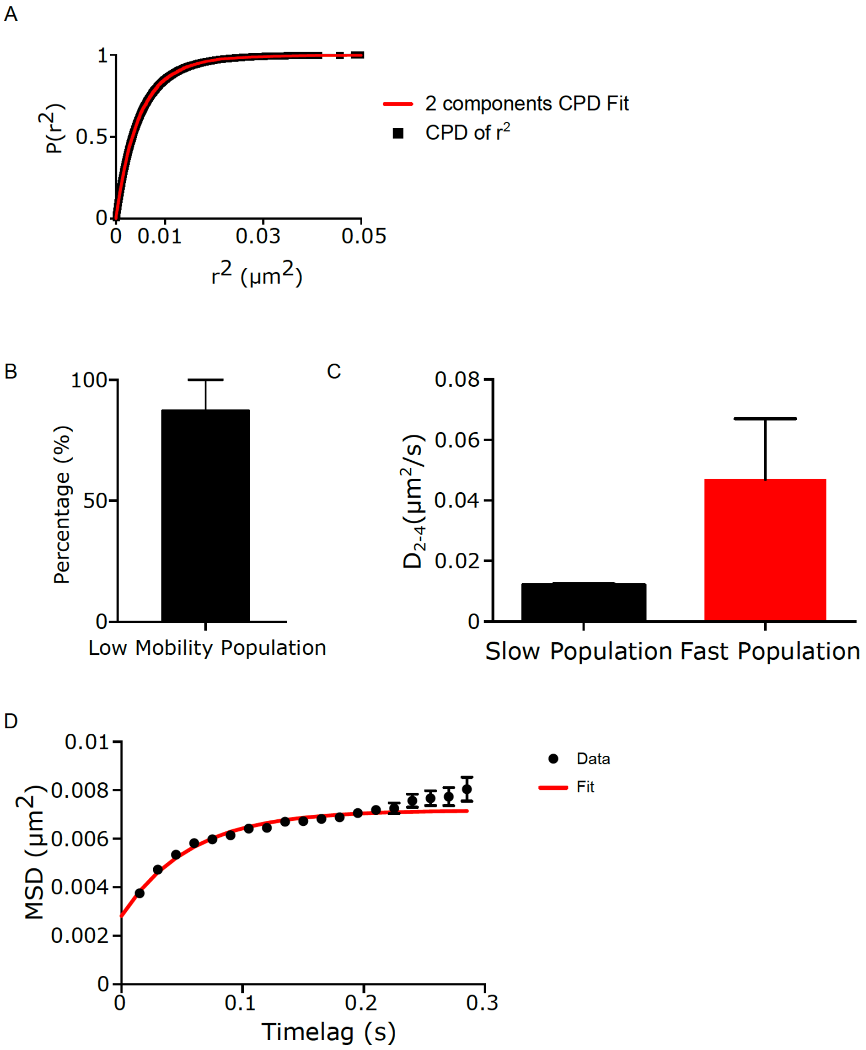

Figure S8. PR trajectories inside condensates exhibit two different populations. (A) Cumulative probability distribution (CPD) function of squared displacements (r^2) is best fitted with a two-population function. (B) Percentage of trajectories belonging to the slow diffusion population. (C) $D_{2\text{-}4}$ values of the slow (black) and fast (red) diffusing populations. (D) Cumulative MSD plot of PR trajectories inside condensates show confined motion (see Methods for details regarding the analysis and fittings).

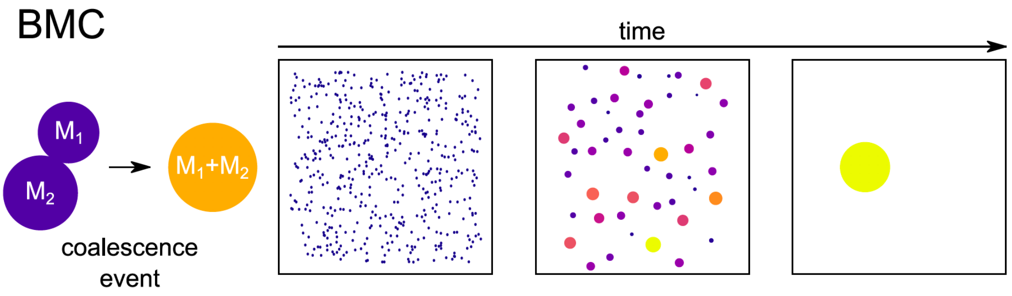

Figure S9: Example of a coalescence event following the standard BMC model. Two condensates with M1 and M2 particles, respectively, coalesce to form a larger one containing M1+M2 particles, considering area conservation in 2D or volume in 3D. Right panels: evolution of a system undergoing standard BMC, in which condensates diffuse following Stokes drag, and coalesce until reaching an equilibrium state with a single condensate containing all the particles. Colors showcase the size of condensates (dark for small and light for big).

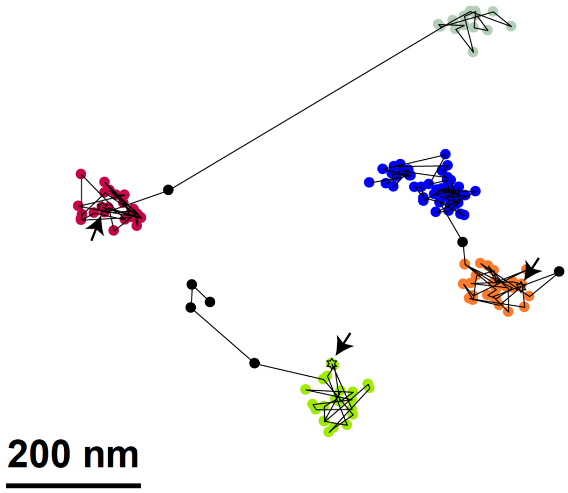

Figure S10. Escaping events in PR trajectories detected by the clustering algorithm. Representative trajectories with escaping events from condensates detected by the clustering algorithm. Condensates are labeled with different colors and the beginning of each trajectory is marked with an arrow.

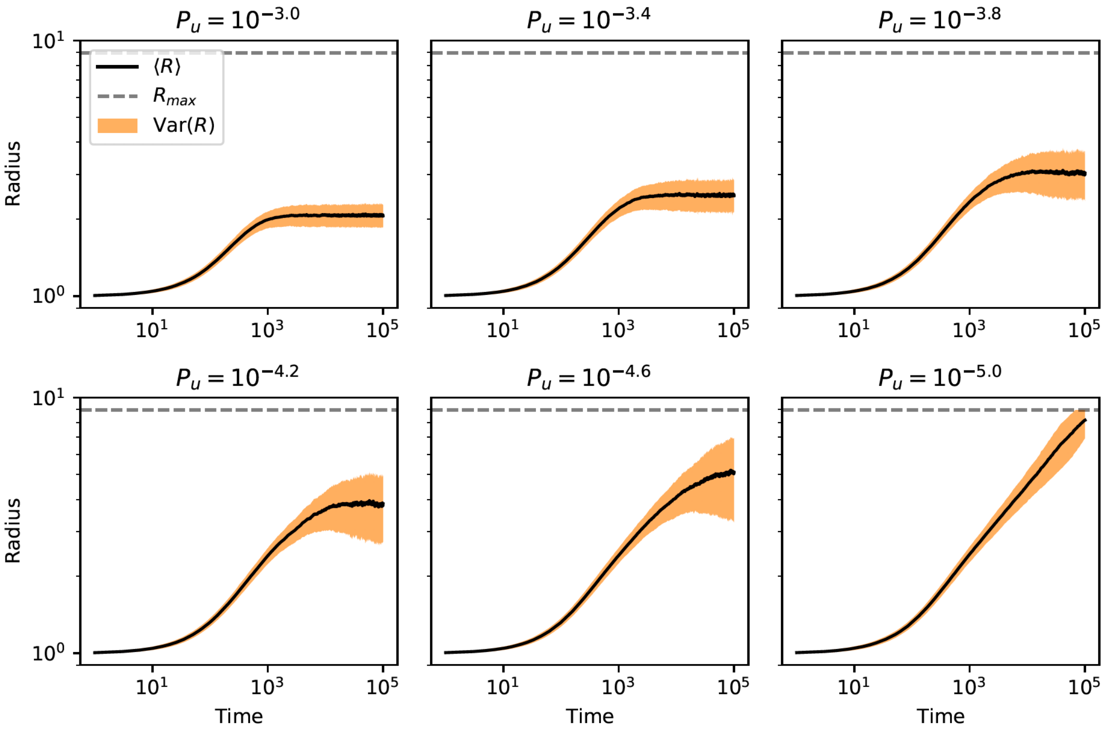

Figure S11: Condensate radius mean (black line) and variance (shaded area) for the same system properties and values of P_u as considered in Fig. 4B.

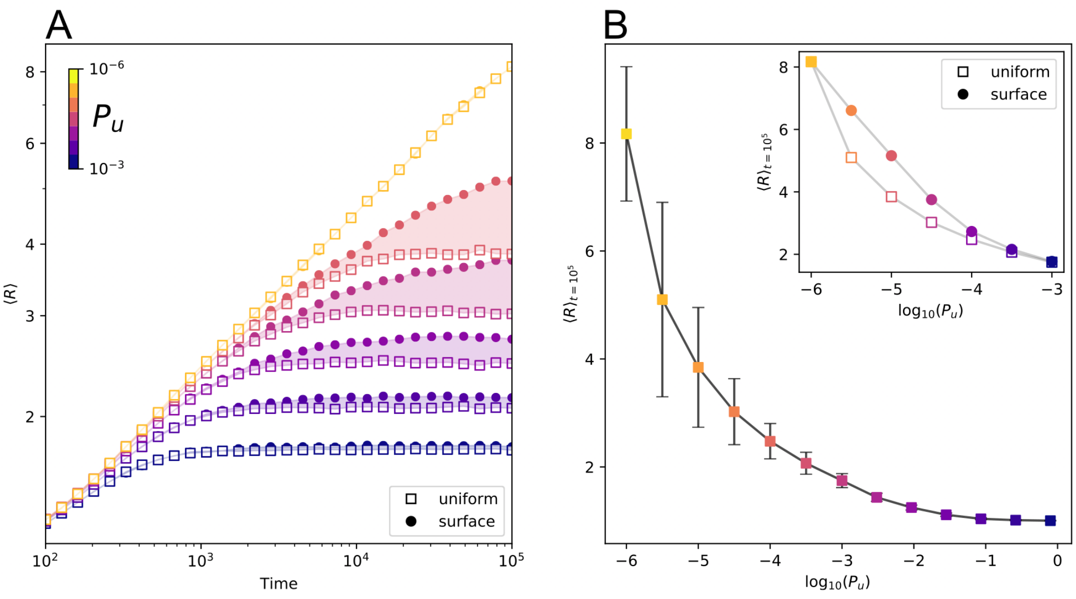

Figure S12. Difference in condensate growth between uniform and surface effects for large values of P_u. (A) Mean radius size for a system with uniform escaping probability (empty squares) and one in which only particles at the boundary can escape (circles), for various unbinding probabilities. (B) Mean radius size at $t = 10^5$ as a function of P_u . Error bars indicate the corresponding deviation. The inset showcases the mean radius size at $t = 10^5$ over the same set of P_u presented in panel (A), for both uniform and surface escaping mechanisms.
